# Case Report of Initial Manifestation of Hypogonadotropic Hypogonadism Based on Unoccluded Growth Plates in a 26-Year-Old Vietnamese Patient

**DOI:** 10.7759/cureus.113283

**Published:** 2026-07-24

**Authors:** Richard Lehnert, Anna-Lena Buschhart, Emanuel Schultz, Stephanie Schneider, Daniel Schrednitzki

**Affiliations:** 1 Orthopedics, Traumatology, Hand- and Reconstructive Surgery, Sana Hospital Lichtenberg, Berlin, DEU

**Keywords:** delayed puberty, gonadotropin-releasing hormone, growth plates, hypogonadotropic hypogonadism, secondary hypogonadism, tibial fracture

## Abstract

Hypogonadotropic hypogonadism is a rare endocrine disorder characterized by deficient secretion of gonadotropin-releasing hormone (GnRH), leading to reduced levels of luteinizing hormone (LH) and follicle-stimulating hormone (FSH), ultimately resulting in impaired sexual maturation and pubertal development. While typically diagnosed during adolescence, rare cases present in adulthood as incidental findings. This case report describes an unusual presentation of hypogonadotropic hypogonadism discovered incidentally during treatment of a traumatic tibial fracture in a 26-year-old male patient, highlighting the importance of recognizing unoccluded growth plates as a potential indicator of underlying endocrine pathology.

A 26-year-old Vietnamese male (height 1.70 m, weight 59 kg, BMI 20.4 kg/m²) presented to the emergency department on December 27, 2022, with left lower leg pain following a work-related accident. Radiographic imaging revealed a spiral fracture of the left tibial shaft with notably unoccluded growth plates. Physical examination demonstrated absent secondary sexual characteristics, including lack of pubic hair and prepubertal genitalia. The patient reported no previous medical conditions or current medications, though his history included mumps at age 10, raising the possibility of orchitis. Serial laboratory measurements over three consecutive days (at 8:45 AM each day) revealed consistently low serum testosterone (15.0-20.0 ng/dL; reference: 249-836 ng/dL), LH (0.17-0.22 IU/L; reference: 1.70-8.60 IU/L), and FSH (0.66-0.69 IU/L; reference: 1.50-12.4 IU/L), confirming the diagnosis of hypogonadotropic hypogonadism. Other pituitary function tests were within normal limits. The tibial fracture was treated with closed reduction and intramedullary interlocking nail fixation. Following endocrinological consultation, the patient was initiated on testosterone replacement therapy administered every three months.

This case demonstrates an uncommon adult presentation of hypogonadotropic hypogonadism diagnosed incidentally following traumatic injury. The presence of unoccluded growth plates on routine radiographic imaging in an adult patient served as a crucial diagnostic clue, emphasizing the importance of systematic evaluation of skeletal maturity and endocrine status when such findings are encountered. Early recognition and appropriate hormonal replacement therapy are essential for addressing both the endocrine deficiency and potential skeletal complications. Long-term follow-up will determine the effectiveness of testosterone therapy and whether growth plate closure can be achieved.

## Introduction

Hypogonadotropic hypogonadism is characterized by deficient or absent secretion of gonadotropin-releasing hormone (GnRH) from the hypothalamus, resulting in reduced luteinizing hormone (LH) and follicle-stimulating hormone (FSH) secretion and, consequently, low testosterone levels and impaired pubertal development. The condition typically presents during adolescence with delayed or absent puberty, lack of secondary sexual characteristics, small testicular volume, decreased libido, and infertility. Skeletal manifestations include delayed bone age, reduced bone mineral density, and persistent open growth plates beyond the expected age of epiphyseal fusion [1]. Congenital hypogonadotropic hypogonadism is a rare disorder, with estimated incidences ranging from approximately 1 in 8,000 to 1 in 40,000 men and a clear male predominance compared with women [2].

In males, testosterone is essential for the pubertal growth spurt, accrual of bone mineral density, and epiphyseal closure, largely through aromatization to estradiol, which mediates fusion of the growth plates. In addition, testosterone drives the development of secondary sexual characteristics, including enlargement of the testes and penis, growth of facial and body hair, deepening of the voice, and increased muscle mass [3]. The progression of pubertal development and testicular growth is therefore routinely assessed using standardized pubertal staging systems such as Tanner stages for genital and pubic hair development [4] and orchidometric measurement of testicular volume. Standard clinical assessment therefore combines biochemical evaluation with systematic examination of secondary sexual characteristics and pubertal staging, for example, using Tanner stages and testicular volume measurement.

Most patients with congenital hypogonadotropic hypogonadism are diagnosed in adolescence, when failure of normal pubertal progression becomes evident. However, some individuals remain undiagnosed until adulthood and may present incidentally during evaluation for unrelated medical conditions [1,5]. In such cases, persistently open growth plates on radiographs represent a highly unusual and strongly suggestive finding of underlying hypogonadism, although other endocrine and systemic disorders can also delay epiphyseal fusion and must be considered in the differential diagnosis [1,6,7].

This case report describes a 26-year-old male in whom hypogonadotropic hypogonadism was first suspected after incidental detection of unoccluded growth plates during workup of a tibial fracture. The report aims to raise awareness of this atypical presentation in adult trauma patients and to highlight the importance of routine assessment of skeletal maturity and secondary sexual characteristics when interpreting radiographs in young adults.

## Case presentation

Patient presentation and history

A 26-year-old Vietnamese man presented to the emergency department of Sana Klinikum Lichtenberg in December 2022, following a work-related accident. The patient reported acute pain in the left lower leg. Due to language barriers (the patient spoke only Vietnamese), all communication was facilitated through Triaphon, a phone-based translation service (Triaphon GmbH, Berlin, Germany).

Physical examination revealed a patient of relatively small stature, 1.70 m tall and weighing 59 kg, corresponding to a BMI of 20.4 kg/m², consistent with a normal weight range without overt signs of malnutrition or obesity. Focused musculoskeletal examination of the left lower extremity demonstrated pain, swelling, and deformity consistent with a tibial shaft fracture. Notably, general physical examination revealed striking findings: complete absence of secondary pubic hair and prepubertal-appearing external genitalia with small testicular volume, features highly suggestive of hypogonadotropic hypogonadism [2]. Pubertal development was not formally staged using the Tanner scale at the time of presentation; however, clinical examination revealed prepubertal external genitalia, absence of pubic hair, and qualitatively small testicular volume, consistent with Tanner stage G1P1 [4].

The patient's medical history was unremarkable, with no reported chronic conditions or regular medications. However, a significant historical detail emerged: the patient had contracted mumps at age 10. This raised clinical concern for possible post-infectious orchitis, which could have contributed to testicular dysfunction, although primary hypogonadism caused by orchitis would typically present with elevated gonadotropins rather than the low levels subsequently observed.

Radiographic findings

**Figure 1:**
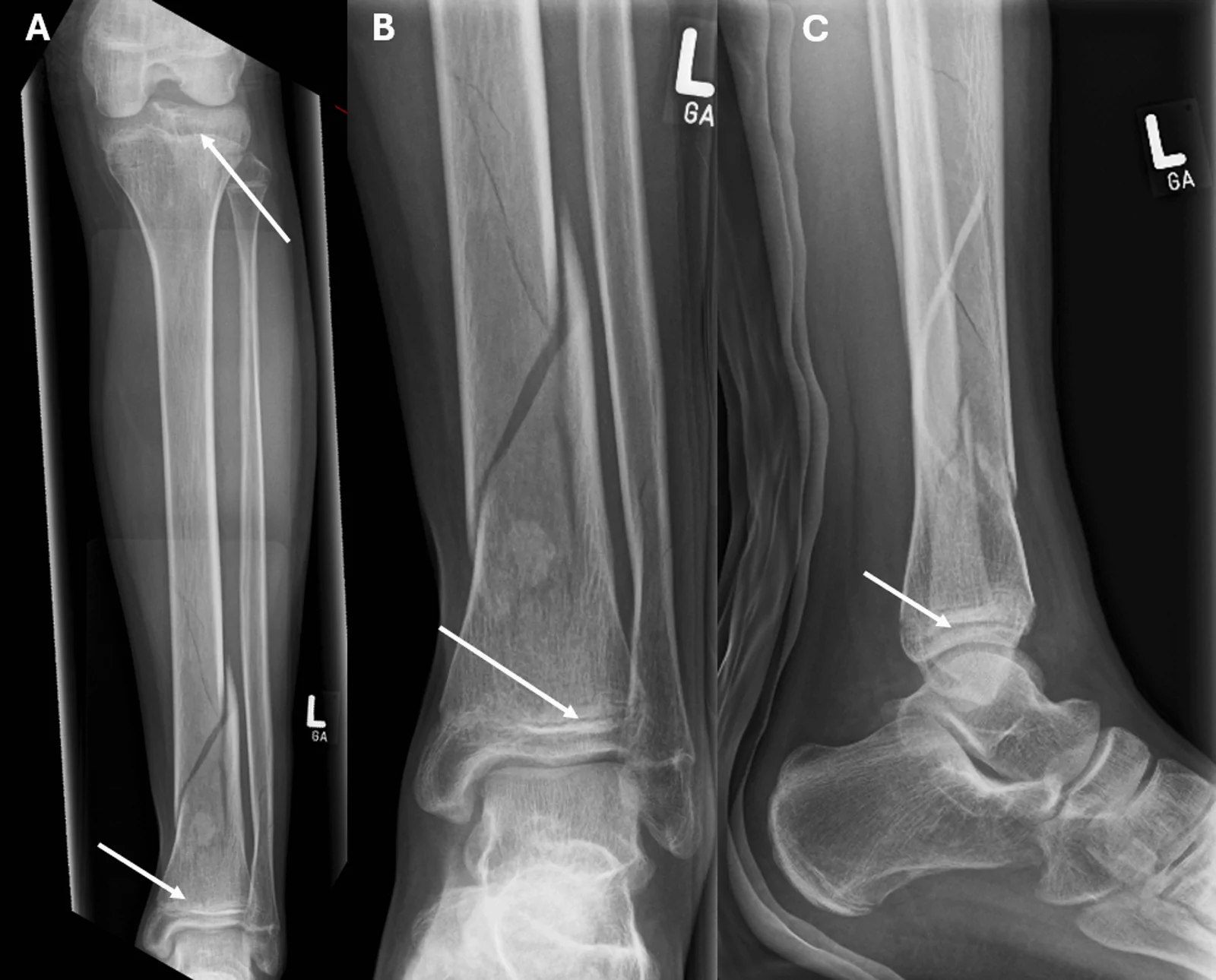
Preoperative radiographs of the left ankle showing a spiral fracture of the tibial shaft with persistently open growth plates (arrows indicate open physes at the proximal and distal tibia). (A) Anteroposterior long view; (B) Anteroposterior short view; (C) Lateral view

Standard anteroposterior and lateral radiographs of the left lower leg revealed a spiral fracture of the tibial shaft. Computed tomography imaging was performed for surgical planning and confirmed the fracture pattern. Remarkably, both radiographic modalities clearly demonstrated unoccluded (open) growth plates at the proximal and distal tibia (Figure 1), a finding distinctly abnormal in a 26-year-old man, as physeal closure typically occurs by age 16-18 in males.

The presence of open growth plates in an adult patient immediately raised suspicion of an underlying endocrine disorder affecting skeletal maturation, prompting further investigation.

Laboratory investigations

Given the suspicious clinical and radiographic findings, comprehensive endocrine evaluation was initiated. Serum samples were collected on three consecutive days, with blood drawn at 8:45 AM each day to control for circadian variation in hormone levels. The following results were obtained:

The hormonal pattern revealed low testosterone levels accompanied by inappropriately low (rather than elevated) gonadotropin levels, confirming secondary (hypogonadotropic) hypogonadism rather than primary testicular failure. Additional pituitary function tests, including thyroid-stimulating hormone (TSH), prolactin, adrenocorticotropic hormone (ACTH), and growth hormone axes, were within normal limits, suggesting isolated gonadotropin deficiency (see Table 1). Other pituitary axes were not systematically evaluated at the time of trauma admission, and free T4, morning cortisol, and insulin-like growth factor 1 (IGF‑1) were not measured, which limits the completeness of the initial endocrine workup.

**Table 1:** Laboratory results demonstrating hypogonadotropic hypogonadism with preserved other pituitary functions

Hormone	Patient Values	Reference Range
Testosterone (ng/dL)	15.0-20.0	249-836
LH (IU/L)	0.17-0.22	1.70-8.60
FSH (IU/L)	0.66-0.69	1.50-12.4
TSH (mIU/L)	Normal	Normal
Prolactin	Normal	Normal
ACTH	Normal	Normal

Diagnosis

Based on the constellation of clinical findings (prepubertal physical appearance, absent secondary sexual characteristics), radiographic evidence (unoccluded growth plates at age 26), and laboratory confirmation (low testosterone with low LH and FSH), the diagnosis of hypogonadotropic hypogonadism was established.

Surgical management

**Figure 2:**
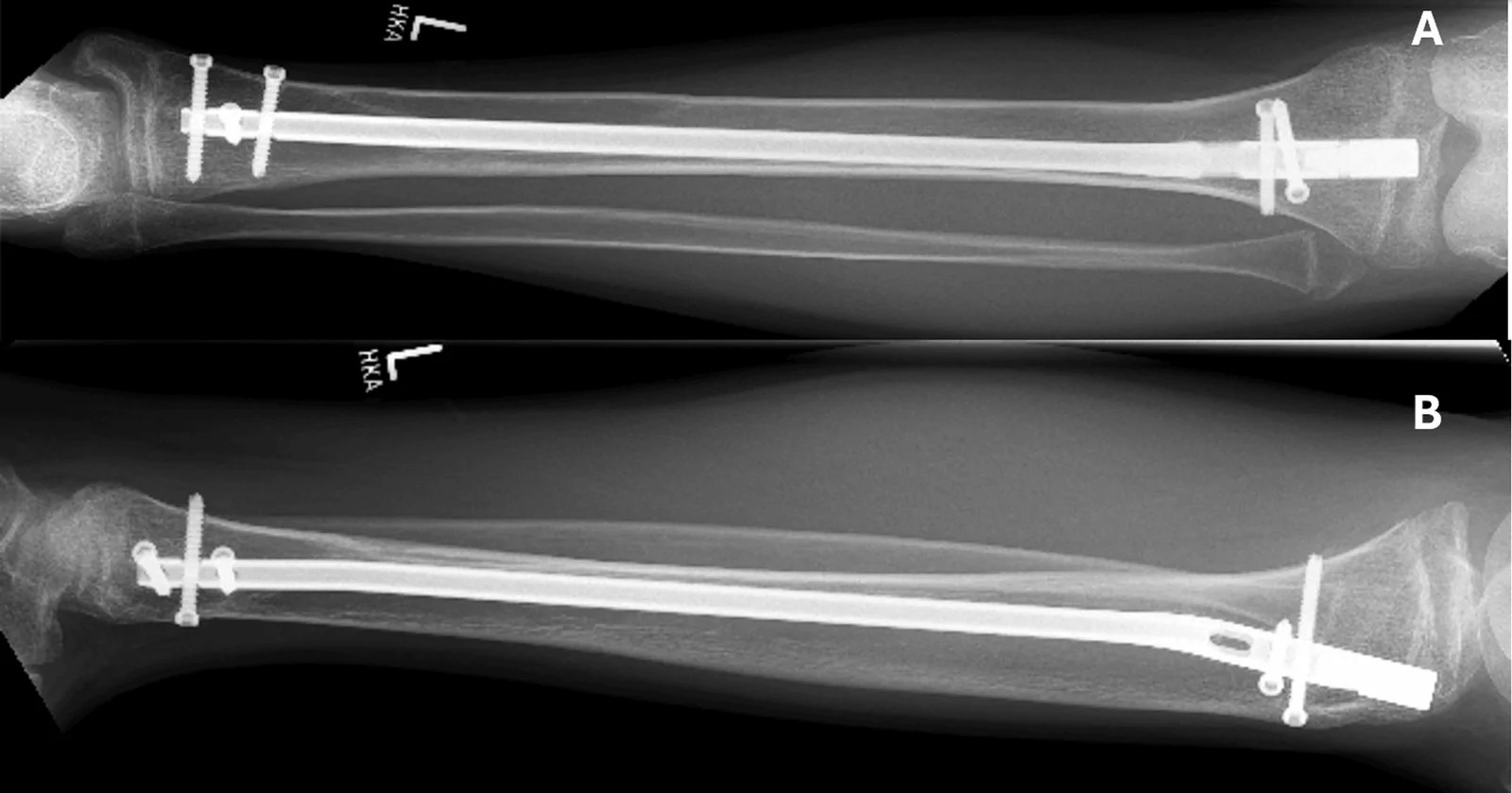
Postoperative radiographs of the left lower leg showing intramedullary interlocking nail fixation with satisfactory fracture alignment and hardware position. (A) Anteroposterior view; (B) Lateral view

The acute tibial fracture required orthopedic intervention. The patient underwent closed reduction and internal fixation with an intramedullary interlocking nail under general anesthesia. The surgical procedure was performed using standard technique with fluoroscopic guidance. Proximal and distal interlocking screws were placed to provide rotational and axial stability (Figure 2). The surgical procedure proceeded without complications. Postoperative radiographs confirmed satisfactory fracture reduction and hardware positioning.

Endocrinological management and follow-up

Following endocrinologic consultation, the patient was initiated on testosterone replacement therapy using intramuscular testosterone undecanoate 1000 mg. Injections were scheduled at baseline, after six weeks, and subsequently every 12 weeks, in line with current recommendations for long-acting testosterone formulations. This regimen was chosen because of its convenient dosing interval, reliable pharmacokinetic profile, and ability to induce stable serum testosterone levels sufficient for virilization.

The therapeutic goals were to induce the development of secondary sexual characteristics, increase muscle mass and bone mineral density, promote growth plate closure, and improve overall metabolic health and quality of life. A structured monitoring plan was established, including regular assessment of serum testosterone levels, hematocrit and hemoglobin, as well as clinical evaluation of virilization, mood, and sexual function. In addition, dual-energy X-ray absorptiometry (DEXA) was planned at baseline and at one- to two-year intervals to monitor bone mineral density. At the time of writing, no follow-up imaging or DEXA measurements are yet available; therefore, any statements regarding growth plate closure and long-term skeletal outcome remain speculative and must be interpreted with caution.

## Discussion

This case represents an unusual presentation of hypogonadotropic hypogonadism, diagnosed incidentally in adulthood during evaluation of acute traumatic injury. Several aspects of this case warrant detailed discussion.

Delayed diagnosis in adulthood

Hypogonadotropic hypogonadism is typically diagnosed during adolescence when patients present with delayed puberty or failure to develop secondary sexual characteristics [5]. The standard diagnostic criterion includes the absence of typical pubertal development by age 18 years [5]. However, in this 26-year-old patient, the condition remained undiagnosed until adulthood--a scenario that, while uncommon, has been reported in medical literature [1]. Possible explanations for delayed diagnosis include limited access to healthcare during adolescence, cultural or social factors affecting medical evaluation, geographic and linguistic barriers (a patient from Vietnam with limited healthcare access), gradual onset minimizing subjective awareness of abnormality, and lack of systematic screening in asymptomatic individuals.

Radiographic findings as a diagnostic clue

Normal skeletal maturation in males involves progressive physeal closure, with most growth plates fusing by late adolescence. The persistence of open growth plates at age 26 is therefore highly unusual and strongly suggestive of longstanding hypogonadism, as adequate sex steroid levels are required for epiphyseal fusion [3]. In this context, unoccluded physes on routine radiographs indicate delayed skeletal maturation and should be recognized as a radiographic clue to underlying endocrine pathology rather than an incidental variant. Persistently open growth plates in adults warrant careful consideration of sex steroid deficiency and other endocrine or systemic disorders that interfere with bone maturation and epiphyseal closure [6,7].

Hormonal pattern and differential diagnoses

The laboratory findings in this case, characterized by consistently low serum testosterone levels in combination with inappropriately low LH and FSH, are typical of secondary (central) hypogonadism rather than primary testicular failure. Primary hypogonadism due to conditions such as Klinefelter syndrome, post-infectious orchitis, or chemotherapy-induced gonadal damage is usually associated with low testosterone in the presence of elevated gonadotropin levels as a consequence of loss of negative feedback. In contrast, secondary hypogonadism is defined by low testosterone together with low or inappropriately normal LH and FSH and reflects impaired hypothalamic-pituitary signalling, as seen in congenital hypogonadotropic hypogonadism, Kallmann syndrome or pituitary lesions [1,6,8,9].

In the present patient, the history of mumps infection at the age of 10 years initially raised concern for post-infectious orchitis and primary testicular damage. However, the hormonal pattern clearly supports central hypogonadism and makes isolated peripheral testicular failure unlikely.

At the same time, the absence of pituitary MRI at the time of trauma care means that structural hypothalamic-pituitary lesions cannot yet be fully excluded, and the presumed diagnosis of isolated GnRH deficiency must be considered provisional pending further imaging and, ideally, genetic testing.

Further diagnostic evaluation would include olfactory testing, as anosmia strongly suggests Kallmann syndrome [2,10,11]. MRI of the hypothalamic-pituitary region is indicated to exclude structural lesions such as pituitary adenomas, craniopharyngiomas, or infiltrative processes, and abnormal findings have been reported in approximately 10-15% of patients with hypogonadotropic hypogonadism [12,13]. In addition, panel-based genetic testing is recommended for comprehensive etiological assessment, as more than 50 genes have been associated with congenital hypogonadotropic hypogonadism and pathogenic variants can be identified in roughly 40-50% of affected individuals [2,12,14], with ANOS1 (Kallmann syndrome 1) representing the most common X-linked form [12]. 

These investigations are consistent with current clinical recommendations for the evaluation and management of Kallmann syndrome and related forms of congenital hypogonadotropic hypogonadism [15].

Pathophysiology and molecular mechanisms

Recent research has highlighted the complex pathophysiology of hypogonadotropic hypogonadism, extending beyond simple GnRH neuronal dysfunction. Smedlund and Hill [3] demonstrated that non-neuronal cells play crucial roles in the development and function of the hypothalamic-pituitary-gonadal axis. Disruption of supporting glial cells, tanycytes, or vascular elements may contribute to GnRH deficiency even in the absence of primary neuronal pathology [3,16]. Current understanding recognizes both congenital and functional forms of hypogonadotropic hypogonadism, with functional forms potentially reversible following correction of underlying metabolic, nutritional, or psychological stressors [16-18]. Notably, approximately 10-20% of patients with apparent congenital hypogonadotropic hypogonadism experience spontaneous reversal of hypogonadism after testosterone therapy discontinuation, suggesting dynamic plasticity of the reproductive neuroendocrine system [18]. This expanding understanding of disease mechanisms may lead to novel therapeutic approaches, including targeted molecular interventions and personalized treatment strategies based on genetic profiles [2,12,16].

Management considerations

The cornerstone of treatment for hypogonadotropic hypogonadism is hormone replacement therapy. Two principal therapeutic approaches are available. Testosterone replacement therapy directly replaces the deficient hormone, thereby inducing virilization and the development of secondary sexual characteristics. Although this treatment does not restore fertility, it effectively addresses the metabolic, skeletal, and psychosexual consequences of hypogonadism [7,8,19]. Alternatively, gonadotropin therapy or pulsatile GnRH therapy stimulates endogenous testosterone production and may preserve or restore fertility. Human chorionic gonadotropin, administered in combination with recombinant FSH or human menopausal gonadotropin, can induce spermatogenesis in 70-90% of patients, with reported pregnancy rates ranging from 40-70%, depending on baseline testicular size and prior treatment history [10,20,21]. Although more complex and costly than testosterone replacement therapy, this approach remains essential for patients seeking fertility.

For the present patient, testosterone replacement therapy was selected as the initial treatment strategy and was administered as an intramuscular injection every three months. This regimen was chosen because of its convenience through quarterly administration, reliable and predictable pharmacokinetic profile, cost-effectiveness, and ability to achieve adequate virilization. In addition, testosterone replacement therapy has been shown to improve bone mineral density and reduce fracture risk in hypogonadal men [7,19].

A critical question in this case is whether testosterone therapy initiated at the age of 26 years will successfully induce growth plate closure. Despite the delayed onset of treatment, exposure to sex steroids generally promotes physeal fusion through the aromatization of testosterone to estradiol, which represents the primary mediator of epiphyseal closure in males [19]. Nevertheless, the ultimate skeletal outcome remains uncertain and will require long-term radiographic follow-up. Should the patient wish to achieve fertility in the future, a transition from testosterone replacement therapy to gonadotropin-based treatment would be necessary. The success of fertility induction is known to correlate inversely with age at treatment initiation and directly with baseline testicular volume [10,20,21].

Fracture management and bone health considerations

The presence of unoccluded growth plates raised theoretical concerns regarding fracture fixation strategy. Open growth plates in skeletally immature patients typically warrant physeal-sparing fixation techniques to avoid premature closure or growth disturbance. However, in this adult patient with pathological growth plate persistence, growth preservation was not a concern; rather, growth plate closure was desirable. Therefore, standard adult fixation with transphyseal intramedullary nailing was appropriate and successfully performed.

An important consideration in patients with hypogonadotropic hypogonadism is the significantly increased risk of osteoporosis and fragility fractures [7,19]. Studies demonstrate that men with untreated hypogonadotropic hypogonadism have markedly reduced bone mineral density, with up to 50% meeting criteria for osteoporosis at diagnosis [7]. The current trauma case, though resulting from acute injury rather than low-energy fragility fracture, emphasizes the importance of comprehensive bone health assessment in all patients with hypogonadism. Testosterone replacement therapy has been shown to significantly improve bone mineral density and reduce fracture risk over 2-3 years of treatment [15]. However, bone density monitoring via DEXA scanning remains essential, particularly in patients diagnosed in adulthood who have missed critical years of peak bone mass accrual during adolescence and early adulthood [7,19].

Clinical implications and learning points

This case highlights several important clinical considerations. First, a systematic assessment of skeletal maturity should be incorporated into the interpretation of radiographs obtained in young adults. The presence of unoccluded growth plates in adulthood should be regarded as a red flag and warrants further endocrine evaluation [6,8]. In appropriate clinical contexts, physical examination should include assessment of secondary sexual characteristics, as these findings may provide important diagnostic clues. Although hypogonadotropic hypogonadism is most commonly diagnosed during adolescence, delayed diagnosis can occasionally result in presentation during adulthood, particularly in individuals with limited access to healthcare or atypical clinical manifestations [6,8,9,21].

The case also underscores the importance of multidisciplinary collaboration between orthopedic surgeons and endocrinologists to ensure comprehensive evaluation and management. Genetic testing should be considered to establish a definitive etiological diagnosis and to facilitate family counseling where appropriate [2,12,14]. Furthermore, long-term monitoring of bone health is essential because patients with hypogonadism are at increased risk of reduced bone mineral density and fragility fractures [7,19]. Psychological support and assessment of quality of life should likewise be integrated into patient management, as delayed diagnosis and pubertal failure are associated with a substantial psychosocial burden [18].

The psychosocial consequences of delayed diagnosis deserve particular attention. Studies involving adults with congenital hypogonadotropic hypogonadism have demonstrated profound effects on self-esteem, body image, sexual function, and interpersonal relationships, with many patients reporting feelings of isolation and inadequacy during adolescence while their peers underwent normal pubertal development [21]. Web-based needs assessments have further identified unmet needs for peer support, accessible educational resources, and specialized psychological services [21]. Recognition of these psychological dimensions is therefore essential for providing holistic, patient-centered care.

Limitations and future directions

This case report has several limitations. Long-term follow-up data regarding the patient's response to testosterone therapy, growth plate closure, and final skeletal outcome are not yet available. In addition, further diagnostic evaluation, including olfactory testing to assess for Kallmann syndrome [14,15], pituitary MRI to exclude structural lesions [12,13], and comprehensive genetic panel testing [2,14], was not performed. At the time of the initial trauma care, no MRI of the hypothalamic-pituitary region had yet been performed. Therefore, we cannot fully exclude a structural central cause, and the presumed diagnosis of isolated GnRH deficiency remains provisional pending further imaging. These investigations would provide valuable etiological information and may contribute to more accurate prognostic assessment. Moreover, the initial endocrine workup did not include measurement of free T4, morning cortisol, or IGF‑1. As a result, subtle impairment of thyroid, adrenal, or growth hormone axes cannot be definitively excluded, and this incomplete hormonal profiling represents an additional limitation of the present case report. In addition, bone age assessment was not performed, which limits our ability to quantify the degree of skeletal delay and to correlate radiographic findings with the patient’s chronological age.

Future follow-up will address several clinically relevant questions. An important objective will be to determine whether testosterone therapy successfully induces growth plate closure, which is expected to occur within approximately 18-36 months according to published data [19]. The timing of physeal fusion following treatment initiation will also be evaluated. Furthermore, longitudinal assessment will determine whether the patient develops complete secondary sexual characteristics, with gradual virilization anticipated over a period of two to three years [6,8]. Additional follow-up will focus on the effects of treatment on bone mineral density and fracture healing, with DEXA scanning recommended at baseline and at one- to two-year intervals [7,19]. Genetic testing may clarify whether the patient harbors identifiable mutations associated with congenital hypogonadotropic hypogonadism [2,12,14]. Monitoring will also assess the possibility of spontaneous reversibility following treatment, a phenomenon reported in approximately 10-20% of affected individuals [18]. Future management decisions may depend on the patient's reproductive goals, as a desire for fertility would necessitate a transition from testosterone replacement therapy to gonadotropin-based treatment [10,20,21]. Finally, ongoing evaluation of quality of life and psychosocial functioning will be important to assess the broader impact of treatment on patient well-being [11].

Systematic prospective data on patients diagnosed with hypogonadotropic hypogonadism in adulthood remain limited. Consequently, this case contributes valuable information regarding the natural history, treatment response, and clinical outcomes of hypogonadotropic hypogonadism when diagnosis occurs after the expected age of pubertal completion [8,9,17].

Clinical message

In everyday practice, persistently open growth plates on radiographs of adult trauma patients should prompt targeted endocrine evaluation, including assessment of gonadal and pituitary hormone levels, and early referral to an endocrinologist. Systematic radiographic assessment of skeletal maturity in younger adults, combined with clinical examination of secondary sexual characteristics, can prevent missed diagnoses of hypogonadism and other endocrine disorders that present incidentally during orthopedic care. Multidisciplinary collaboration between orthopedic surgeons, radiologists, and endocrinologists is essential to ensure that such abnormal findings lead to appropriate diagnostic workup and long‑term management.

## Conclusions

For orthopedic surgeons and emergency physicians, this case highlights that unoccluded growth plates in adult patients represent a rare but important radiographic red flag that requires prompt endocrinological investigation and careful differential diagnosis rather than a pathognomonic finding on its own. Early recognition and appropriate hormonal replacement therapy are essential for addressing endocrine deficiency and supporting skeletal maturation. Long-term follow-up in this patient will be necessary to determine the effectiveness of testosterone therapy and to provide further insights into skeletal outcomes when treatment is initiated in adulthood.
